# Menstrual cup acceptability and functionality in real‐world use: A cross‐sectional survey of young people in Australia

**DOI:** 10.1111/ajo.13910

**Published:** 2024-12-13

**Authors:** Julie Hennegan, Ana Orozco, Alexandra Head, Jennifer L. Marino, Yasmin Jayasinghe, Megan S. C. Lim

**Affiliations:** ^1^ Maternal, Child and Adolescent Health Program Burnet Institute Melbourne Victoria Australia; ^2^ Melbourne School of Population and Global Health University of Melbourne Melbourne Victoria Australia; ^3^ Murdoch Children's Research Institute Melbourne Victoria Australia; ^4^ Department of Obstetrics, Gynaecology, and Newborn Health Royal Women's Hospital and University of Melbourne Melbourne Victoria Australia; ^5^ Specialty of Child and Adolescent Medicine The University of Sydney School of Clinical Medicine Sydney New South Wales Australia; ^6^ Royal Children's Hospital Melbourne Victoria Australia; ^7^ School of Population Health and Preventive Medicine Monash University Melbourne Victoria Australia

**Keywords:** consumer product safety, gynaecology, menstrual health, menstrual hygiene products, women's health

## Abstract

**Background:**

Menstrual cups offer a cost‐effective and environmentally sustainable product for many young people. While clinical trials have shown their safety and effectiveness, no studies have investigated their performance in real‐world use.

**Aims:**

To describe the acceptability and functionality (continuation, discomforts, leakage, and adverse events) of menstrual cups and investigate the supportive role of product knowledge.

**Materials and Methods:**

A cross‐sectional online survey of 530 people aged 15–24 living in Australia who had ever used a menstrual cup, recruited via a menstrual cycle tracking application.

**Results:**

More than half of participants (55%) were still using their first menstrual cup at the time of the survey, 16% had switched to an alternative cup, and 29% had discontinued use. In their first cycle of use, 54% of participants reported leakage and 25% reported pain or discomfort with the cup in place. Many participants (45%) reported being unable to remove the cup on their first attempt, with subsequently 17% requiring help to remove it, and 2% reported displacement of an intra‐uterine device during removal. These figures decreased for subsequent cycles. Half of the participants were aware prior to using a cup that different cup models may offer a better fit for different individuals. This knowledge was associated with decreased odds of needing help to remove the cup (adjusted odds ratio 0.57, 95% CI 0.35–0.94) or discontinuing use (adjusted relative risk ratio 0.66, 95% CI 0.44–1.00).

**Conclusions:**

Difficulties using menstrual cups are common in real‐world use and higher than reported in clinical trials. Improved education provision may support more positive user experiences.

## INTRODUCTION

Menstrual cup use has rapidly increased.^1^ The bell‐shaped device, made of medical grade silicone, is inserted into the vagina to collect menstrual loss. It is then emptied, sterilised, and can be reused for several years with some manufacturers stating ten years with adequate care. Concerns about the environmental sustainability of single‐use menstrual pads and tampons,^2^ cost pressures,^3^, ^4^, ^5^ and demand for a diverse range of menstrual technologies may all have contributed to increased use in high‐income countries.^1^, ^6^ Two studies on prevalence of menstrual cup users have been reported. In 2021, in Victoria, Australia, 17% of 15–29‐year‐olds reported using a menstrual cup during their last menstrual period,^6^ and in Spain 47% of 18–25 year olds reported use in the past 12 months.^7^

The cost savings and longevity of menstrual cups are also attractive to governments and non‐governmental organisations seeking to provide free or subsidised menstrual products. Across high‐, middle‐, and low‐income countries, there is increased recognition that individuals face difficulties affording menstrual products,^3^, ^8^, ^9^, ^10^, ^11^ prompting initiatives to improve access.^12^, ^13^, ^14^ However, lack of research to inform their use in such initiatives, and inadequate information provision about menstrual cups has meant government efforts have focused on single‐use products.^12^, ^15^, ^16^

A 2019 systematic review of trials, clinical reports and qualitative research found menstrual cups were safe and effective.^17^ Studies within the review focused on participants who had been provided with a menstrual cup by investigators, to test product features or the impacts of providing products on reproductive health.^17^ Many had samples of less than 100 participants, investigated only a single type of cup, and all included education or training as part of delivery.^17^

More evidence to understand menstrual cup user experiences is needed to support informed product choice for individuals and the integration of menstrual cups in product provision initiatives. There is sparse data on discomforts and adverse events among menstrual cup users, with no studies capturing real‐world use. The role of education and importance of menstrual cup ‘fit’, such as the specific size, shape and firmness, for user satisfaction is unclear.

In this study we aimed to describe the acceptability and functionality (continuation, discomforts, leakage, and adverse events) of menstrual cups in real‐world use among young people in Australia. Further, we sought to determine the potential role of product knowledge to improve acceptability.

## MATERIALS AND METHODS

This study is reported according to STROBE guidelines.^18^

### Data collection

Participants were recruited through the menstrual cycle tracking application *Clue*.^19^ In‐app pop‐up messages were displayed to 111 142 users in the target age bracket between August and November 2022. Pop‐ups linked to the study information and survey hosted on REDCap.^20^ All materials were in English and the survey took approximately 15 min to complete. Participants could enter a draw to win one of three AUD$100 vouchers.

### Participants

Eligible participants were aged 15–24 years, resided in Australia, had a menstrual period in the past three months and had ever tried using a reusable menstrual product (menstrual cup, reusable menstrual underwear, or reusable pad). Participants were provided with an information sheet and indicated their consent. Before consenting, participants aged 15–17 years were required to correctly complete a comprehension form to assess their understanding of the study and capacity to provide informed consent. We sought to recruit a sample of 2000 reusable product users. A total of 500 users of any product type would provide precision in prevalence estimates (eg a prevalence of 20% would have a confidence interval of 17–24%) and 80% power to detect a 10% difference in proportions.

### Variables and measurements

The complete questionnaire is provided in Appendix S1.

#### Demographic characteristics

Participants self‐reported their socio‐demographics. Postcodes were used to determine Australian Bureau of Statistics' remoteness areas^21^ and index of relative socio‐economic advantage and disadvantage classifications.^22^

#### Knowledge prior to use

Participants were asked if they had sufficient information to make an informed choice of cup, and the sources of information for use. They were asked to indicate different characteristics of their first menstrual cup (eg shape, size), which included an ‘I don't know’ option. As an indicator of knowledge, we asked if participants were aware prior to use that different menstrual cup characteristics may be better suited to different individuals; ‘no’ and ‘not sure’ options were combined.

#### Acceptability and functionality

Continuation, switching, and discontinued use were self‐reported. Participants were asked about their motivations for discontinuation or switching, experience of using an alternative cup, and their confidence in cup use.

To assess discomforts, leakage and adverse events, participants were asked if they experienced any of a list of 13 difficulties or discomforts during the first cycle of menstrual cup use, their second to sixth cycle of use, and after six cycles. These options were reduced (eg discomfort inserting and discomfort removing were combined) into seven categories for analysis.

#### Menstrual cup use

Participants reported their pattern of menstrual cup use, as well as any alteration of the stem, and use during urination and defecation.

#### Open text survey responses

Following questions capturing discomforts, participants were presented with an optional open text question: ‘Is there anything else you'd like to tell us about experiencing discomforts or difficulties related to using a menstrual cup?’

### Data analysis

Analyses were completed using Stata 17. Descriptive statistics present participants' knowledge, experiences and self‐reported acceptability, leakage, and safety. Participants were free to skip any question or stop the survey at any time, and not all questions were relevant to all respondents (eg discomforts experienced in the second cycle of menstrual cup use only applied to those using the cup for at least two cycles). Complete case analysis is presented for each question with missing responses excluded from denominators. Bivariate and multivariable logistic regressions were used to test associations between knowledge about product fit prior to use and acceptability and safety during the first cycle of use, with multinominal logistic regression used to test the association between these predictors and cup continuation. No correction was made for multiple comparisons. Multivariable analyses were adjusted for socio‐demographics that were associated with product knowledge in binary logistic regressions (*P* < 0.10).

Qualitative content analysis was used to analyse participant open text responses. After familiarisation with the responses, inductive codes were generated and refined through discussion, capturing participants' further explanations about the discomforts or concerns experienced.

### Ethics approval

Ethics approval was given by the Alfred Health Research Ethics Committee (project number 275/22).

## RESULTS

### Respondents

Among the 1988 survey participants, 530 who had ever used a menstrual cup and responded to questions regarding cup acceptability and functionality were included in this study.

Participant demographics, characteristics of acquiring a menstrual cup, and purchase‐related knowledge are displayed in Table 1.

**Table 1:** Participant demographics, including the number of missing cases, and acquiring a menstrual cup (*N* = 530)

Demographic characteristic	*n* (%)
Age	
15–19	167 (31.6)
20–24	362 (68.4)
*Missing*	*1*
Socio‐economic index of advantage and disadvantage quintile	
Lowest	40 (8.4)
Second	47 (9.9)
Third	65 (13.7)
Fourth	94 (19.8)
Highest	228 (48.1)
*Missing*	*(56)*
Remoteness	
Major Cities Australia	368 (77.6)
Inner Regional Australia	82 (17.3)
Outer Regional Australia	20 (4.2)
Remote and very remote	4 (0.8)
*Missing*	*(56)*
Gender identity	
Female	492 (93.7)
Non‐binary/gender fluid	31 (5.9)
Male	2 (0.4)
*Missing*	*(5)*
Country of birth	
Australia	427 (80.9)
Other	101 (19.1)
*Missing*	*(2)*
Identify as Aboriginal and/or Torres Strait Islander	
No	512 (96.6)
Yes	14 (2.6)
*Missing*	*(4)*
Educational level (current or completed)	
School	150 (28.7)
Tertiary	373 (71.3)
*Missing*	*(7)*
Currently working	
No	104 (20)
Yes	417 (80)
*Missing*	*(9)*
Lives with (multi‐response)	
Alone	39 (7.4)
With parents	277 (52.3)
With partner	119 (22.5)
Friends or housemate	112 (21.1)
Children	13 (2.5)
Money for recreational purposes	
<AU$120 per week	437 (85)
≥AU$120 per week	77 (15)
*Missing*	*(16)*
Years since first acquiring menstrual cup	
<1	85 (16.1)
1 year	129 (24.4)
2 years	124 (23.5)
3+ years	190 (36.0)
*Missing*	*(2)*
How first menstrual cup was acquired	
Purchased by participant	404 (76.2)
Purchased by someone else (on participant request)	81 (15.3)
Other (received as gift, receive in free promotion, borrowed)	45 (8.5)
Had enough information to make an informed choice about which menstrual cup to purchase	
Strongly disagree	32 (6.1)
Disagree	168 (31.9)
Agree	225 (42.7)
Strongly agree	102 (19.4)
*Missing*	*(3)*
Before using a cup, was aware that menstrual cups have different characteristics and may offer better fit for different individuals	
Yes	232 (43.8)
No	266 (50.2)
Not sure	32 (6.0)

Italics denotes missing. Missing not included in reported proportions.

### Knowledge prior to use

As displayed in Table 1, many participants did not have enough information to make an informed choice of products and were unaware of differing cup characteristics. Furthermore, many reported not knowing the characteristics of their menstrual cup, including firmness (20.3%, *n* = 107), model size (13.1%, *n* = 69), or cervix height (47.0%, *n* = 248). Many others selected the most common options for firmness (medium, 55.1%, *n* = 291), size (medium 39.8% *n* = 210, small 42.8% *n* = 226) and cervix height (regular model 46.0%, *n* = 243).

For guidance on insertion and removal, most participants used information provided on the product box (76.8%, *n* = 407) and/or information online (68.5%, *n* = 363). Fewer consulted family (9.3%, *n* = 49) or friends (8.7%, *n* = 46), and 2.0% (*n* = 11) received information from their school or a health care provider.

### Acceptability and functionality

#### Continuation

Half (49.6%; *n* = 259/522) of participants were still using their first menstrual cup at the time of the survey, and 5.9% (*n* = 31/522) were using a replacement cup of the same model. Among these, 75.1% (*n* = 215) had been using their cup for longer than six cycles, with 24.9% (*n* = 71) for less than six. A further 15.5% (*n* = 81) of participants were using an alternative model of menstrual cup. Of these participants, 60.3% (*n* = 47) switched within the first year of use.

A total of 28.9% (*n* = 151/522) of participants had discontinued using a menstrual cup. Among this group, 40.0% (*n* = 58/145) discontinued after the first cycle, 43.5% (*n* = 63/145) after two to six cycles, and 16.6% (*n* = 24/145) after six cycles of use. Of participants who had discontinued using a cup, 68.6% (*n* = 98/143) did so due to discomforts or leakage, while a smaller percentage (22.4%; *n* = 32/143) cited inconvenience changing, cleaning, or preferring other products, and 9.1% (*n* = 13/143) due to other reasons (eg changes to blood flow).

#### Switching

Among 232 participants who no longer use a menstrual cup or had switched to a different cup from their first one, 123 (54.7%) reported that they had tried just one additional type of menstrual cup, while 85 (37.8%) had tried two cups and 17 (7.6%) tried three.

Among the 81 (15.5%) participants who switched to an alternative cup, 35.1% (*n* = 27/77) reported this significantly improved their experience, 40.3% (*n* = 31/77) reported it somewhat improved their experience, 16.9% (*n* = 13/77) reported their experience remained the same, and 7.8% (*n* = 6/77) reported a worse experience with the new cup.

#### Discomfort, leakage, and adverse events

Only 10.4% (*n* = 55/530) of participants successfully inserted their menstrual cup on the first attempt. Most (50.1%) (*n* = 265/530) inserted after two‐to‐three attempts, while 30.1% (*n* = 160/530) required four or more attempts, and 9.4% (*n* = 50/530) never successfully inserted the cup.

Among participants who had used a menstrual cup for more than one cycle, 23.5% (*n* = 97/413) reported they were still not confident or never confident using their cup, 47.7% (*n* = 197/413) reported feeling confident after one to three cycles, and 20.8% (*n* = 86/413) after four to six cycles.

Table 2 presents further participant‐reported product difficulties.

**Table 2:** Self‐reported menstrual cup discomforts, leakage, and adverse events during the 1 cycle, 2–6 cycles and >6 cycles of use of the first menstrual cup used, and during use of a second type of menstrual cup

	First menstrual cup						Subsequent menstrual cups			
	1st cycle of use		2nd–6th cycle of use		>6th cycle		1st cycle of use		2nd–6th cycle	
	*n* = 490	%	*n* = 418	%	*n* = 279	%	*n* = 76	%	*n* = 64	%
Did not experience any listed discomforts, leakage, or adverse events	30	6.1	60	14.3	113	40.5	23	30.3	30	46.9
Acceptability										
Discomfort inserting or removing	425	86.7	272	65.1	62	22.2	33	43.4	9	14.1
Unable to remove on first attempt	221	45.1	190	45.4	107	38.3	34	44.7	20	31.2
Required help to remove	82	16.7	60	14.3	28	10.0	10	13.2	7	11.0
Pain or discomfort when cup *in situ*	120	24.5	76	18.2	33	11.8	14	18.4	5	7.8
Leakage										
Cup leaked while in use	262	53.5	120	28.7	33	11.8	15	19.7	5	7.8
Adverse events										
Displacement of intra‐uterine device	12	2.4	0	0	0.01	0.4	1	1.3	0	0.0

Number of participants responding changes based on the number of cycles they used a menstrual cup, and if they reported ever using an alternative cup. Participants could select multiple options.

A total of 145 participants (27.4%) provided open text responses elaborating on the difficulties and discomforts listed in our survey. Table 3 provides illustrative quotations contextualising the difficulties indicated in the quantitative data. Only four participants raised issues not captured in the survey, reporting infections or urinary issues they believed were related to the cup. Notably, 19% (*n* = 27) of open text responses described the perception that discomfort or pain was due to a lack of information, and in 20% (*n* = 29) of responses participants reflected that additional knowledge and practice using the cup improved their experience.

**Table 3:** Open text survey responses highlighting the varied severity of discomforts experienced and user perceptions

Discomforts or difficulties	Participant open text responses
Discomfort inserting or removing a menstrual cup	‘The first time trying to pull it out was scary because the grip is very different to a tampon. feel like it is stuck and you won't ever get it out… all my friends have had same first scary experience but then afterwards it's fine!’ ‘One issue was not being able to remove it… felt like my fingers weren't long enough to reach each side to clamp it together to reach the suction. Took myself and my partner 30 mins of attempts to finally get it’ ‘I had sex before the first time using a menstrual cup so I knew my body pretty well and had used tampons as well but using a menstrual cup, just getting it right (especially getting it out the first time was challenging… my dad was a doctor so, his help was fine but I cannot imagine how some girls deal with this alone…esp when Google can be very unhelpful)’
Pain or discomfort when cup *in situ*	‘I kept being told that the cup was a one size fit all and I really hurt myself trying to fit it in all the time and feeling anxiety that I was different somehow’ ‘It's not all the time that there is discomfort, but it does happen and in the first day or two it can make my cramps worse’ ‘It's just difficult to get it far up enough, I can still feel the end of it in my vagina’
Leakage	‘My pelvic muscles emptied the cup, moved it and made it ineffective I want to be able to use my menstruated cups but the risk of leaking makes me too anxious to wear it during the day and during the night’ ‘I sometimes get some leakage on my underwear overnight on the first or second night so I have started wearing liners or pads as well to prevent leaking which means I'm still using single use items’
Safety concerns	‘Had a lot of urinary issues due to lack of sterilisation because I was not informed of it’ ‘Something about this cup makes me more susceptible to thrush and other bacterial infections in my vagina which is very uncomfortable whilst on my period’
Role of education and learning	'While I did research about the cups themselves I don't think there was much information about what to expect in terms of sensation or any possibility of discomfort’ ‘Practice made perfect for me, trial and error really helped in getting comfortable. I also have a lower cervix on the third or fourth days of my cycle, so the cup feels uncomfortable, like its sitting too low, so I usually don't use one on those days’ ‘I found the first cup I used was too long for my cervix height and the stem would chafe my vulva due to how much it would stick out, even after I cut it back it did this and cause a lot of discomfort. This led me to get another cup that was more suited to my cervix height and was a lot more comfortable, I don't even notice it when it's in now’

### Associations between knowledge and experiences

Older age (odds ratio (OR) = 1.54, 95% CI 1.07–2.24) and being born outside Australia (OR = 2.45, 95% CI 1.55–3.87) were associated with increased odds of knowing prior to using a menstrual cup that different cup characteristics may offer the best fit for different individuals (Appendix S2). This knowledge was not associated with experiencing discomforts, leakage, or intra‐uterine device (IUD) displacement. However, prior knowledge of cup characteristics was associated with reduced odds of requiring help to remove the cup during the first cycle of use, and discontinuing using a menstrual cup, after adjustment for age and country of birth (Table 4). These associations were robust to adjustment for all sociodemographic characteristics (Appendix S2).

**Table 4:** Associations between pre‐use knowledge of cup characteristics and experience of acceptability, leakage, adverse events, and discontinuation during the first menstrual cycle of use (*N* = 487)

	Did not have knowledge prior to selection *n* (%)	Knowledge prior to selection *n* (%)	OR (95% CI)	aOR (95% CI)^†^
	*n* = 238	*n* = 249		
Did not experience any listed discomforts, leakage or adverse issues	14 (5.9)	16 (6.4)	1.10 (0.52, 2.30)	
Acceptability				
Discomfort inserting or removing	205 (86.1)	217 (87.1)	1.09 (0.65, 1.84)	
Unable to remove on first attempt	115 (48.1)	114 (47.9)	0.79 (0.55, 1.13)	
Required help to remove	51 (21.43)	31 (12.5)	0.52 (0.32, 0.85)*	0.57 (0.35, 0.94)*
Pain or discomfort when cup *in situ*	61 (25.6)	59 (23.7)	0.90 (0.59, 1.36)	
Leakage				
Cup leaked while in use	134 (56.3)	127 (51.0)	0.81 (0.56, 1.15)	
Safety				
Displacement of intra‐uterine device	6 (2.5)	6 (2.4)	0.95 (0.30, 3.00)	
Discontinuation^‡^			RRR (95% CI)	aRRR (95% CI)
Continued using cup	133 (51.2)	157 (60.6)	1.00	
Discontinued	83 (31.9)	67 (25.9)	0.68 (0.46, 1.01)**	0.66 (0.44, 1.00)*
Switched brands and continued use	44 (16.9)	35 (13.5)	0.67 (0.41, 1.11)	0.68 (0.41, 1.13)

* *P < 0.05*.

** *P* = 0.060.

^†^ With adjustment for age and country of birth.

^‡^ This was asked as a separate question to other discomforts and the total number responding was 519, including 290 who continued using the same cup (reference group).

OR, odds ratio; RRR, relative risk ratio; aOR, adjusted odds ratio; aRRR, adjusted relative risk ratio.

### Menstrual cup use

A third (31.3%, *n* = 158/480) of participants reported trimming their menstrual cup's stem. Of these, 86.0% (*n* = 135) reported this improved comfort.

Among participants, 77.7% (*n* = 258/332) always felt comfortable urinating with their cup *in situ*. Fewer participants (39.9%, *n* = 131/328) always felt comfortable defecating with the cup inserted, with 12.7% (*n* = 42/330) reporting they always removed their cup to defecate, 45.2% (*n* = 149/330) sometimes removed it, and 42.1% (*n* = 139/330) never removed it.

## DISCUSSION

This study captured reports of menstrual cup acceptability and safety during real‐world use. We found a similar proportion of our sample continued using a menstrual cup (71%) compared to the pooled prevalence identified in a 2019 systematic review of menstrual cup trials (73%).^17^ In our sample this included 16% who had switched to an alternative cup type or brand. Menstrual cup‐related discontinuation was much higher than reported in past trials (pooled, 11%), with 69% of our participants discontinuing use due to discomfort and 22% due to cup‐related inconvenience. We also found a substantially higher prevalence of discomforts reported by our sample than in clinical trials.^17^ In the first cycle of use, 87% reported discomfort inserting or removing the cup. Almost half (45%) reported being unable to remove the cup on the first attempt, and 17% reported needing assistance. In contrast, a pooled 9% reported difficulty removing when assessed in clinical trials (pooled *n* = 461).^17^ In open text, participants described the distress associated with this experience, and the need for greater education and support.

Participant characteristics, knowledge and training on cup use, the varied cup types used, and access to support were all likely to differ in real‐world use and contribute to the higher observed prevalence of discomforts in our sample compared to clinical trials.^17^, ^23^ Many participants reported having insufficient information about using a menstrual cup. Pre‐use awareness that different menstrual cups may fit better for different individuals was associated with lower odds of discontinuation and requiring help to remove the cup (indicative of a more severe difficulty). This suggests that experiences using menstrual cups may be improved with education for young people. Such resources must be provided prior to uptake, as initial negative experiences see many young people abandon the product. Difficulties attenuated over time, and half of those who continued felt confident using their menstrual cup after three cycles. A quarter of our sample reported cup leakage during the first cycle, reducing to 12% after six cycles.

Education and support will also be essential for the inclusion of menstrual cups in initiatives to improve the affordability or accessibility of menstrual products. In Australia, the distribution of free single‐use menstrual products in public‐funded schools is most often delivered through vending machines,^15^ with initiatives for distribution to the broader public also focused on this mechanism.^14^ While vending machines may offer accessible and private access to products, they do not facilitate the education that would be needed to expand provision to include reusable products. Similarly, non‐governmental organisations providing menstrual products globally must be aware of the potential discomforts and difficulties associated with menstrual cups, ensure monitoring for adverse outcomes, and provide education and support. Further, self‐reported use of products in our sample suggests many young people are not comfortable defecating with menstrual cups *in situ*, which may impact the duration throughout the day for which they can be used without the need for removal.

Our findings add to the evidence since the systematic review^17^ that menstrual cups present a risk for IUD expulsion, with 12 participants reporting this occurred on their first cycle of use. We did not ask participants to report contraceptive methods, so we are unable to assess what proportion of those using an IUD this represents. A recent online survey in the United States reported that 9% of IUD users reported at least one expulsion due to a menstrual cup,^24^ and a further trial observed increased accidental self‐removal of an IUD.^25^

Recruitment through the *Clue* app^19^ allowed us to access a large sample of young people, and avoided the potential bias of a publicly available online survey which may be shared through social networks with less representative experiences of menstrual cup use. However, *Clue* users have been found across the United States, South Africa and India to reflect younger, more educated and urban populations.^26^ We have also previously found that more advantaged groups report greater uptake of reusable menstrual products.^6^ The implications of this bias for our results suggest that the level of knowledge to support menstrual cup use among the full population, and potential extent of discomforts, may be greater than we have reported here. It is also plausible that participants with more positive or more negative experiences of menstrual cups were motivated to respond to the survey advert, and to complete the full set of survey questions.

In conclusion, menstrual cups continue to represent a cost and environmentally friendly menstrual product option. However, the extent of difficulties and discomforts that may be experienced, particularly during initial menstrual cycles of use, mean more comprehensive information and support are urgently needed to support the increasing number of young people adopting this product.^1^, ^6^ Product provision policies and initiatives must incorporate education and support if seeking to incorporate menstrual cups into delivery, and should continue providing other product options alongside cups. More research to understand optimal cup fit for individual users is needed, to further support informed choice.

## FUNDING

This study was funded through an internal Burnet Institute seed funding award and the National Health and Medical Research Council (NHMRC) (GNT2008600, GNT1166356, GNT1161445, MRFAR000308). JH time on this work was funded by the Reckitt Global Hygiene Institute (RGHI). The views expressed are those of the authors and not necessarily those of RGHI. ML is supported by a NHMRC Career Development Fellowship. JLM is supported by NHMRC Project Grant GNT1161445. YJ is supported by Medical Research Future Fund MRFAR000308.

## Supporting information


**Appendix S1.** Survey including questions focused on menstrual cup use. Any queries email the corresponding author.

**Appendix S2.** Bivariate associations between pre‐use knowledge of cup characteristics and socio‐demographics during the first menstrual cycle of use.
