# Supplementary material for: Menstrual cup acceptability and functionality in real‐world use: A cross‐sectional survey of young people in Australia
Source: Aust N Z J Obstet Gynaecol. 2024 Dec 13;65(3):382–9. doi: 10.1111/ajo.13910 (PMC12282031; doi:10.1111/ajo.13910)
Supplement: Supplementary file 1 — Appendix S1. Survey including questions focused on menstrual cup use. Any queries email the corresponding author. [file AJO-65-382-s002.docx]

**Supplementary Material 1:** survey including questions focused on menstrual cup use. Any queries email the corresponding author.

Reusable Menstrual products: Experiences and choices survey

The questions below were filterable based on respondents answers. Respondents only answered questions corresponding to one product. Therefore, the full length of the survey does not indicate an individual respondents survey questions.

| **Variable name** | **Question** | **Coding categories** | **Show if** |
| --- | --- | --- | --- |
|  | **Identification – RE Completes** |  |  |
|  | **Consent** |  |  |
|  | [online consent form]  Has the participant provided consent in the online form? | Yes  No | **Survey ends if no consent** |
|  | **Eligibility** |  |  |
|  | Have you ever tried using a reusable menstrual product (menstrual cup, period underwear, reusable pads)? | Yes  No [not eligible for study]  I do not wish to say [not eligible for study] |  |
| **A** | **Demographics**  These first questions are about you and your background, and about your life at home. | |  |
| **age** | What is your age? | Drop down list with ages 15-25 |  |
| **postcode** | What is your current postcode?  Write 99 if you do not wish to say. |  |  |
| **gender** | What is your current gender identity?  (please select all that apply) | Male 1  Female 2  Non-binary/gender fluid 3  My gender is not listed,  please specify 4  I do not wish to say -99 |  |
| **country** | What country were you born in? | Australia 1  Other 2  I do not wish to say -99 |  |
| **atsi** | Are you of Aboriginal or Torres Strait Islander origin? | No 0  Yes, Aboriginal 1  Yes, Torres Strait Islander 2  Yes, Aboriginal and  Torres Strait Islander 3  I do not wish to say -99 |  |
| **student** | Are you currently studying? | Yes 1  No 0  I do not wish to say -99 |  |
| **study_level** | At what level are you currently studying? | High school, year 10 or lower 1  High school, year 11 2  High school, year 12 3  TAFE, college or diploma 4  University – undergraduate course 5  (e.g. Bachelor degree)  University – postgraduate course 6  (e.g. Masters, Doctorate)  I do not wish to say -99 | If student==1 |
| **study_highest** | What is the highest level of education you have completed? | High school, year 10 or lower 1 High school, year 11 2 High school, year 12 3 TAFE, college or diploma 4 University – undergraduate course 5  (e.g. Bachelor degree) University – postgraduate course 6  (e.g. Masters, Doctorate) I do not wish to say -99 | If student!=1 |
| **work** | Are you currently working? | Yes 1  No 2  I do not wish to say -99 |  |
| **work_hours** | How many hours do you spend working outside the home in an average week? | [open text] | **Set for 1-3 digit number response** |
| **livewith** | Who do you live with currently?  (Please select all that apply) | Alone   1  Parent(s)/Guardian 2  Siblings(s) 3  My partner   4  Friend(s)/housemate(s)  5  My child(ren)   6  Other family  7  I do not wish to say  -99 |  |
| **Livewith_no** | How many of the following do you live with?   - Females - Males - Non-binary persons | [open text] I do not wish to say  -99 | **Set for 1-3 digit number response** |
| **money** | During a normal week, how much money do you have to spend on yourself for recreational purposes? | Less than $40 1  $40–$79 2  $80–$119 3  $120–$199 4  $200–$299 5  $300 or over 6  I do not wish to say -99 |  |
| **menarche** | At what age did you experience your first menstrual period? | [dropdown list of ages 5-25] |  |
| **b** | **General: access to information and first use of menstrual materials** |  |  |
|  | These first few questions are for you to tell us about your first experiences in hearing about various menstrual products and when you tried them for the first time. | |  |
| b1 | How old were you when you first used the following menstrual products?   - Disposable pads - Tampons - Reusable menstrual underwear - Reusable pads - Menstrual cups/disc | Under 10 1  Individual years 10-25  I do not wish to say -99 |  |
| b | **Menstrual cups: access to information and decision making** |  |  |
|  | This next set of questions will ask you about where you first heard and learnt about menstrual cups and what influenced or informed your decision-making process when selecting a cup, if you made that choice. |  |  |
| b3 | What sources have you used for information about menstrual cups? | Family 1  Friends 2  In a class at school  (e.g., sex ed, health etc) 3  From a health care professional  (doctor or nurse) 4  Online advertising 5  Social media  (e.g., Facebook, Instagram etc) 6  Educational website 7  Other, please specify 8  I do not wish to say -99 |  |
| b5 | What motivated you to try using a menstrual cup?  Please select your **top two** motivations from the list. | Cost efficiency 1  Environmental/sustainable 2  Friends/family recommended 3  To learn about own menstrual  Cycle 4  Comfort or mobility advantages 5  Health benefits 6  I do not wish to say -99 |  |
| b6 | How did you get your first menstrual cup? | I purchased it myself 1  Someone purchased it for me,  on my request 2  Someone purchased it for me  (as a gift or suggestion for use) 3  I got it for free (promotional) 4  I borrowed it 5  Other, please specify 6  I do not wish to say -99 |  |
| b7 | Before using your first menstrual cup, did you know that different menstrual cup brands have different characteristics and may be a better fit for different people? | Yes 1  No 2  Not sure 3  I do not wish to say -99 |  |
| b8 | Before you purchased your first menstrual cup, did you compare different cup brands/types? | Yes, online 1  Yes, in store 2  No 3I do not wish to say -99 | **b6= 1 OR 2** |
| b8_sources | What information sources (such as websites) did you use to compare menstrual cups/discs? | [open text] | **If b8=1** |
| b9 | Did you considering the following characteristics about your body and menstrual cycle before selecting your menstrual cup?  *Please select all that apply* | None of the below 0  Your age 1  your cervix height 2  Your pelvic floor strength 3  Your ‘flow’  (how heavy/light your period is) 4  Your activity level 5  Having given birth 6  Incontinence 7  If you have an IUD 8  Other, please specify 9  I do not wish to say -99 | **b8= 1 OR 2** |
| b10 | Is there anything else you would like to share about selecting your first menstrual cup? | [open text] | **b6= 1 OR 2** |
| b11 | Please select the characteristics of the first menstrual cup you used.  Refer to the images as required.  If you are not sure of the characteristic select ‘I do not know.’ | - Shape   - V   - Bell   - Round   - Asymmetrical   - Disc   - Other, please specify   - I do not know   - I don’t wish to say - Firmness   - Hard   - Medium   - Soft   - I do not know   - I don’t wish to say - Model (size)   - Small   - Medium   - Large   - I do not know   - I do not wish to say - Model (cervix height)   - High cervix or long vaginal canal   - Low cervix or short vaginal canal   - Regular model   - Other, please specify   - I do not know   - I do not wish to say | **Revert to all respondents** |
| b12 | What is the name/brand of the cup you selected? | [open text] |  |
| b13 | Where did you get information about how to insert and remove your menstrual cup?  *Please select all that apply.* | Family 1  Friends 2  A teacher or in a Class at school 3  A health care professional 4  (doctor or nurse)  Online videos or article 5  Packaging on the box 6  I taught myself with no other input 7  I do not wish to say -99 |  |
| b14 | Thinking about the first menstrual cup you used, select the answer that most matches your feelings about the below sentences:   - I had enough information to make an informed choice about which menstrual cup to buy - I was satisfied with the choice that I made in purchasing my first menstrual cup | Strongly Agree 1  Agree 2  Disagree 3  Strongly disagree 4  I do not wish to say -99 | **B6=1 OR 2** |
| C | **Menstrual cups: experiences and challenges of use** |  |  |
|  | The next set of questions will ask you about your experiences and challenges using your menstrual cup for the first time, and with later use. | |  |
| c1 | How long after you got your first menstrual cup did you try to use it for the first time? | First cycle after getting it 1  Within 2 – 3 cycles/months 2  Within 4 – 6 cycles/months 3  Within 6-11 cycles/months 4  Over 1 year 5  I still do not use my cup 6  Not sure/can’t remember 7  I do not wish to say -99 |  |
| c2 | Approximately how many attempts did it take you to successfully insert your first menstrual cup?  *That is, you were able to get the cup inside your vagina, to open into its correct form and to collect your menstrual fluid.* | First attempt 1 2-3 tries 2 4-6 tries 3 6+ attempts 4 Never successfully inserted 5 I do not wish to say -99 |  |
| c3 | Do you still use the first cup you got? | Yes 1  No 0  I do not wish to say -99 |  |
| c4 | Do you still use any menstrual cup? | Yes, at least once  in the last 3 cycles 1  No 0  I do not wish to say -99 | **If C3==0** |
| c5 | Is it the same type/brand as your first cup? | Yes, replacement cup  of the same type and brand 1  No, a different cup 0  I do not wish to say -99 | **If C4=1** |
| c5_why | Why did you replace your first cup?  *Please select all that apply.* | Cup was lost 1  Cup broke or was damaged 2  Cup was old  and wanted to replace 3 | **If c5=1** |
| C5_dif | What motivated you to try a different model or brand of menstrual cup to your first cup?  *Select all that apply* | - Difficulties inserting - Difficulties removing - Cup leaking - Cup felt too hard - Cup felt too soft - Urinary incontinence or feeling the need to urinate - Discomfort related to cervix height or position - Wanted to see if an alternative cup was more comfortable - Wanted to see if an alternative cup performed better - I do not wish to say | **If c5=0** |
| c6 | How long have you been using your menstrual cup? | 1 cycle 1  2-6 cycles 2  6+ cycles to 1 year 3  1-3 years 4  3+ years 5  I do not wish to say -99 | **If C3=1** |
| c7 | How long did you use your first menstrual cup? | 1 cycle 1  2-6 cycles 2  6+ cycles to 1 year 3  1-3 years 4  3+ years 5  I do not wish to say -99 | **If C4=0**  **If C5=0** |
| c7_alt | How long have you used your menstrual cup (including the first cup you used and the replacement cup)? | 1 cycle 1  2-6 cycles 2  6+ cycles to 1 year 3  1-3 years 4  3+ years 5  I do not wish to say -99 | **If C5=1** |
| c8 | For how long in total have you used any subsequent menstrual cup(s)? | 1 cycle 1  2-6 cycles 2  6+ cycles to 1 year 3  1-3 years 4  3+ years 5  I do not wish to say -99 | **If C5=0** |
| c9 | The next question asks about difficulties or discomforts that some people have reported experiencing when using a menstrual cup.  Thinking about the **first** menstrual cup you used  **Please record whether you experienced this:**   1. **During the 1^st^ cycle** 2. **During the 2^nd^-6^th^ cycle of cup use (if c7 or c8 >1 month)** 3. **After the first 6 cycles of use (if c7 or c8 >6 cycles)** | - Difficulty when inserting - Difficulty when removing - Leakage onto underwear - Leakage onto outer clothes or bedding - Pain due to the cup while it was inserted - Feeling the frequent need to urinate due to the cup - Urinary incontinence due to the cup - Nausea - Unable to remove cup on the first attempt - Unable to remove cup and required help to remove - Unable to remove cup and required medical assistance - Displacement of IUD - Other discomfort experienced, please specify - I do not wish to say | **During the 1^st^ cycle**  **C7=1**  **During the 2^nd^-6^th^ cycle of cup use**  **(if c7 or c8 >1 month)**  **After the first 6 cycles of use (if c7 or c8 >6 cycles)** |
| c10 | How many different menstrual cups have you tried? | Drop down 1-10+ | **If C4=0**  **OR 5=0** |
| c11 | Thinking about any menstrual cups that you have tried since your first cup, please record whether you experienced any of the following difficulties or discomforts   1. During the first 6 cycles 2. After the first 6 cycles (only show if C8>6 cycles) | - Difficulty when inserting - Difficulty when removing - Leakage onto underwear - Leakage onto outer clothes or bedding - Pain due to the cup while it was inserted - Feeling the frequent need to urinate due to the cup - Urinary incontinence due to the cup - Nausea - Unable to remove cup on the first attempt - Unable to remove cup and required help to remove - Unable to remove cup and required medical assistance - Displacement of IUD - Other discomfort experienced, please specify - I do not wish to say | **If C4=0 OR C5=0**  **(show >6 months if C8>6 cycles)** |
| c12 | Is there anything else you’d like to tell us about experiencing discomforts or difficulties related to using a menstrual cup? | [optional open text] |  |
| c13 | Did you trim the stem on your menstrual cup? (or on any menstrual cup you have used) | Yes 1  No 0  I do not wish to say -99 |  |
| c14 | Did this improve comfort using the cup? | Yes 1  No 2  I do not wish to say -99 | **If c14=1** |
| c16 | Please select the characteristics of the alternative menstrual cup you used.  If you tried multiple alternatives, please select the characteristics of the alternative you felt worked *best* for you.  If you are not sure of the characteristic select ‘I do not know.’ | - Shape   - V   - Bell   - Round   - Asymmetrical   - Disc   - Other   - I do not know   - I don’t wish to say - Firmness   - Hard   - Medium   - Soft   - I do not know   - I don’t wish to say - Model (size)   - Small   - Medium   - Large   - I do not know   - I do not wish to say - Model (cervix height)   - High cervix or long vaginal canal   - Low cervix or short vaginal canal   - Regular model   - Other, please specify   - I do not know   - I do not wish to say | **C5=0** |
| c17 | Did an alternative menstrual cup work better for you? | Yes, significantly better 1  Yes, somewhat better 2  About the same 3  No, it is worse 4  I do not wish to say -99 |  |
| c18 | Is there anything else you would like to tell us about your motivations for trying different cups or experiences trying different cups? | [open text] |  |
| c19 | What was the main reason (from the below list) you stopped using your cup? | - Too difficult to insert - Too difficult to remove - Uncomfortable when inserted - Painful when inserted - Felt the need to urinate frequently with cup in place - Too inconvenient to change - Too messy - Felt disgusted/dirty dealing with menstrual blood - Preferred using disposable products - Preferred using alternative reusable product - Too difficult to sterilize - Cup was lost or damaged and did not replace it - Changes to menstrual blood flow - Other, specify - I do not wish to say | **If C4=0** |
| c20 | How regularly do you [did you] use your menstrual cup? | I use my cup every period 1  I use my cup some periods 2  I use my cup rarely 3  I do not wish to say -99 | Every |
| c21 | What days/times during your cycle do you [did you] typically use your menstrual cup?  *Select all that apply* | The heavy days of my period 1  The moderate days of my period 2The light days of my period 3  Days when I am at home  Days when I am outside my home  (e.g., to school, work) 4  During exercise 5  While swimming 6  Overnight on heavy days 7  Overnight on light days 8  I do not wish to say -99 |  |
| c22 | When using your menstrual cup, do you [did you] use it in combination with other products? | I use my cup without  other products  I wear a reusable pad/period  undies when using my cup 2  I wear a disposable pad/liner  when using my cup 3  Other, please specify 4  I do not wish to say -99 |  |
| c23 | How many cycles did it take for you to feel confident using a menstrual cup? | I am still not confident/  was never confident 1  1 – 3 cycles 2  4 – 6 cycles 3  6-11 cycles 4  4. Over 12 cycles/ 1 year 5  6. Not sure/can’t remember 6  I do not wish to say -99 |  |
| c24 | How often do you [did you] empty your menstrual cup on a typical day of your period? | 1-2 hrs 1  3-4 hrs 2  5-6 hrs 3  7-8 hrs 4  9-10 hrs 5  11-12 hrs 6  12+ hrs 7  I do not wish to say -99 |  |
| c25 | Do you [did you] feel comfortable urinating (weeing) with your menstrual cup in place | Always 1  Sometimes 2  Never 3  I do not wish to say -99 |  |
| c26 | Do you [did you] feel comfortable defecating (pooing) with your menstrual cup in place | Always 1  Sometimes 2  Never 3  I do not wish to say -99 |  |
| c27 | Do you [did you] remove your menstrual cup to defecate (poo)? |  |  |
| c27 | How often do you [did you] empty (or remove and reinsert) your menstrual cup while you are away from your home (for example at school, university/TAFE, your workplace)? | Never 1  Rarely (less than 6 monthly) 2  Occasionally  (less than every second period) 3  One or two days of my period 4  Every day of my period 5  I don’t use my cup way from home 6  I do not wish to say -99 |  |
| c28 | Do you [did you] delay emptying your menstrual cup while you are away from your home? | Often 1  Sometimes 2  Never 3  I do not wish to say -99 |  |
| c29 | Why do you [did you] delay emptying your cup when away from home? | [open text] |  |
|  | ***End of survey*** |  |  |
