# Supplementary material for: Menstrual cup acceptability and functionality in real‐world use: A cross‐sectional survey of young people in Australia
Source: Aust N Z J Obstet Gynaecol. 2024 Dec 13;65(3):382–9. doi: 10.1111/ajo.13910 (PMC12282031; doi:10.1111/ajo.13910)
Supplement: Supplementary file 2 — Appendix S2. Bivariate associations between pre‐use knowledge of cup characteristics and socio‐demographics during the first menstrual cycle of use. [file AJO-65-382-s001.docx]

**Supplementary Material 2.** Bivariate associations between pre-use knowledge of cup characteristics and socio-demographic during the first menstrual cycle of use.

|  | | **Knowledge prior to selection %(n)** | **Did not have knowledge prior to selection %(n)** | **OR** | **(95%CI)** |
| --- | --- | --- | --- | --- | --- |
| Age (n=529) | 15-19 | 57.5(96) | 71(42.51) |  |  |
|  | 20-24 | 46.7(169) | 193(53.31) | **1.54** | (1.07,2.24) |
| SEAD - quintile (n=474) | Lowest | 55(22) | 18(45) |  |  |
|  | Second | 42.6(20) | 27(57.45) | 1.65 | (0.71,3.86) |
|  | Third | 47.7(31) | 34(52.31) | 1.34 | (0.61,2.96) |
|  | Fourth | 45.7(43) | 51(54.26) | 1.45 | (0.69,3.05) |
|  | Highest | 51.8(118) | 110(48.25) | 1.14 | (0.58,2.24) |
|  | *Missing* | *(22)* | (29) |  |  |
| Remoteness (n=474) | Major cities Australia | 51.1(188) | 180(48.91) | 0.73 | (0.48,1.13) |
|  | Regional Australia | 43.4(46) | 60(56.6) |  |  |
|  | *Missing* | *(22)* | *(29)* |  |  |
| Gender Identity (n=525) | Female | 49.4(243) | 249(50.61) | 1.39 | (0.68,2.84) |
|  | Non-female | 57.6(19) | 14(42.42) |  |  |
| Country (n=525) | Australia | 54.3(232) | 195(45.67) |  |  |
|  | Other | 32.7(33) | 68(67.33) | **2.45** | (1.55,3.87) |
| Educational level (current or completed) (n=523) | School | 52.7(79) | 71(47.33) |  |  |
|  | Tertiary | 49.6(185) | 188(50.4) | 1.13 | (0.77,1.65) |
| Money for recreational purposes (n=514) | <$120 per week | 50.6(221) | 216(49.43) |  |  |
|  | >= $120 per week | 52(40) | 37(48.05) | 0.95 | (0.58,1.54) |

Associations between pre-use knowledge of cup characteristics and experience of acceptability, leakage, adverse events, and discontinuation during the first menstrual cycle of use (n=487).

|  | Did not have knowledge prior to selection  n (%)  N=238 | Knowledge prior to selection  n (%)  N=249 | OR(95%CI) | aOR (95%CI)^1^ | Sensitivity analysis^d^  aOR (95%CI) |
| --- | --- | --- | --- | --- | --- |
| Did not experience any listed discomforts, leakage or adverse issues | 14 (5.9) | 16 (6.4) | 1.10(0.52,2.30) |  |  |
| Acceptability |  |  |  |  |  |
| Discomfort inserting or removing | 205 (86.1) | 217 (87.1) | 1.09(0.65,1.84) |  |  |
| Unable to remove on first attempt | 115 (48.1) | 114 (47.9) | 0.79(0.55,1.13) |  |  |
| Required help to remove | 51 (21.43) | 31 (12.45) | **0.52(0.32,0.85) ^a^** | **0.57(0.35,0.94) ^a^** | **0.60 (0.36-1.00)** |
| Pain or discomfort when cup in situ | 61 (25.63) | 59 (23.69) | 0.90(0.59,1.36) |  |  |
| Leakage |  |  |  |  |  |
| Cup leaked while in use | 134 (56.3) | 127 (51) | 0.81(0.56,1.15) |  |  |
| Safety |  |  |  |  |  |
| Displacement of IUD | 6 (2.52) | 6 (2.41) | 0.95(0.30,3.00) |  |  |
| Discontinuation ^c^ |  |  | RR (95%CI) | aRRR (95%CI) | aRRR (95%CI)^d^ |
| Continued using cup | 133 (51.15) | 157 (60.62) | 1.00 |  |  |
| Discontinued | 83 (31.9) | 67 (25.9) | **0.68(0.46,1.01) ^b^** | **0.66 (0.44,1.00) ^a^** | **0.63 (0.42-0.97)** |
| Switched brands and continued use | 44 (16.9) | 35 (13.5) | 0.67(0.41,1.11) | 0.68 (0.41,1.13) | 0.61 (0.36-1.04) |

***^1^*** with adjustment for age and country of birth.  **^a^** *p <0.05,* **^b^** p =0.060, **^c^** This was asked as a separate question to other discomforts and the total number responding was 519, including 290 who continued using the same cup (reference group)

^d^ with adjustment for age, country of birth, and additional sociodemographics: SEAD-quintile, gender identity, education level and money for recreational spending.
